# Combined maternal risk factors and the Quadruple test to predict late-onset preeclampsia in pregnant Thai women

**DOI:** 10.1186/s12884-023-05623-4

**Published:** 2023-04-22

**Authors:** Phattarika Bunyapipat, Ninlapa Pruksanusak, Chitkasaem Suwanrath, Alan Geater

**Affiliations:** 1 Department of Obstetrics and Gynecology, Faculty of Medicine, Prince of Songkla University, Hat Yai, Songkhla, Songkhla 90110 Thailand; 2 Epidemiology Unit, Faculty of Medicine, Prince of Songkla University, Hat Yai, Thailand

**Keywords:** Late-onset preeclampsia, Quadruple test, Serum inhibin A

## Abstract

**Background:**

This study aimed to evaluate the predictive power of a model combining maternal risk factors and the Quadruple screen test for late-onset preeclampsia (PE).

**Methods:**

All pregnant women that received the Quadruple test for Down syndrome at 15^+ 0^-20^+ 6^ weeks’ gestation were recruited. Maternal serum α-fetoprotein, β-human chorionic gonadotropin, unconjugated estriol, and inhibin A were measured as multiples of the median. A logistic regression model was used to identify predictors associated with late-onset PE with severe features. The receiver operating characteristic (ROC) curve and area under the curve (AUC) were used to assess the model’s predictive ability.

**Results:**

Fifty-five of the 2,000 pregnant women had PE, and 31 of 55 women had late-onset PE. Multivariate analysis identified maternal age ≥ 35 years, inhibin A, history of previous PE, history of infertile, cardiac disease, chronic hypertension, and thyroid disease as significant risk factors. The area under the curve of the receiver operating characteristic curve was 0.78. The likelihood ratio to predict late-onset PE was 49.4 (total score > 60).

**Conclusions:**

Our model combining serum inhibin A with maternal risk factors was useful in predicting late-onset PE. Close monitoring of these patients is recommended.

## Background

Preeclampsia (PE) is a complication that affects 2–8% of all pregnancies and is an important cause of maternal and perinatal morbidity and mortality worldwide [1, 2]. Over the past decade, it has become widely accepted that the pathogenesis of early-onset and late-onset PE differs [3–8], with early-onset PE (before 34 weeks) having a compromised terminal villi volume and surface area, resulting in a smaller and higher infarction of the placenta when compared with late-onset PE. For late-onset PE the thinking is that it results from an imbalance between the cardiovascular supply of pregnant woman and the metabolic demands of the fetuses, and their placenta in the third trimester period. Therefore, early-onset PE is more commonly associated with more severe adverse maternal and neonatal outcomes than late-onset PE [4, 9, 10]. Several studies have investigated first trimester gestation to predict early-onset PE via maternal characteristics, Doppler ultrasound, and maternal serum biochemical substances [11–21]. Early detection can identify the requirement for early prescription of low-dose aspirin, for high-risk pregnant patients before 16 weeks’ gestation. This is in accordance with the standard recommendations for the prevention of PE proposed by the National Institute for Health and Care Excellence (NICE) [22], the American College of Obstetricians and Gynecologists (ACOG) [23], and the International Federation of Gynecology and Obstetrics (FIGO) [24].

Although early-onset PE is associated with more severe maternal and perinatal.

morbidity and mortality, the vast majority of PE cases are late-onset type [25, 26]. Our institute, Songklanagarind Hospital, a referral center in southern Thailand, has a total PE incidence of 3.63%, with 1.26% and 2.37% being early- and late-onset type, respectively [27]. The morbidity and mortality associated with late-onset PE patients more commonly affect mothers than neonates. In low- and middle-income countries, where access to facilities such as specialized medical care and transportation is often limited, 10–15% of direct maternal deaths are associated with PE with severe features and eclampsia [28, 29]. Moreover, severe morbidities, including renal failure, stroke, cardiac arrest, adult respiratory distress syndrome, coagulopathy, and hepatic failure, have been reported in these patients [30, 31]. Although low-dose aspirin might not have a preventive effect on late-onset PE [23, 24], the identification of high-risk patients will result in benefits from close monitoring and early diagnosis and management in order to reduce maternal morbidity and mortality. Over the past decade, several studies have demonstrated the predictive power of combined models for late-onset PE prediction using maternal risk factors, mean arterial pressure, pregnancy-associated plasma protein A, placental growth factor, and uterine artery pulsatility index. These screening models reported a detection rate for late-onset PE of around 33.5–41.3% in the first trimester. However, there are two major concerns when using these tests in low- and middle-income countries; firstly, these tests are often beyond the capacity or accessibility of health services, and secondly, most women do not receive prenatal care until their second trimester or later in their pregnancy [32].

In Thailand, the Quadruple (Quad) test is widely used for Down syndrome screening in the second trimester of gestation. The test consists of four biochemical hormones, namely inhibin A, alpha-fetoprotein (AFP), human chorionic gonadotropin (hCG), and unconjugated estriol (uE3). Several previous studies [33–39] have reported that abnormal Quad test results could predict the detection of high-risk PE patients. Although abnormal serum levels in the Quad test might be associated to PE, neither abnormal serum levels nor maternal risk factors alone resulted in a high detection rate of late-onset PE [22–24, 27, 32].

To our knowledge, no previous studies have used both maternal risk factors and the serum Quad test to improve the detection of PE patients, especially in those with late-onset type, in low- and middle-income countries with limited access to high-technology healthcare, funds, and specialists. Therefore, this study aimed to evaluate the efficacy of combined serum biochemical hormones (the Quad test) and maternal risk factors during the second trimester of pregnancy in predicting late-onset PE.

## Methods

**Fig. 1:**
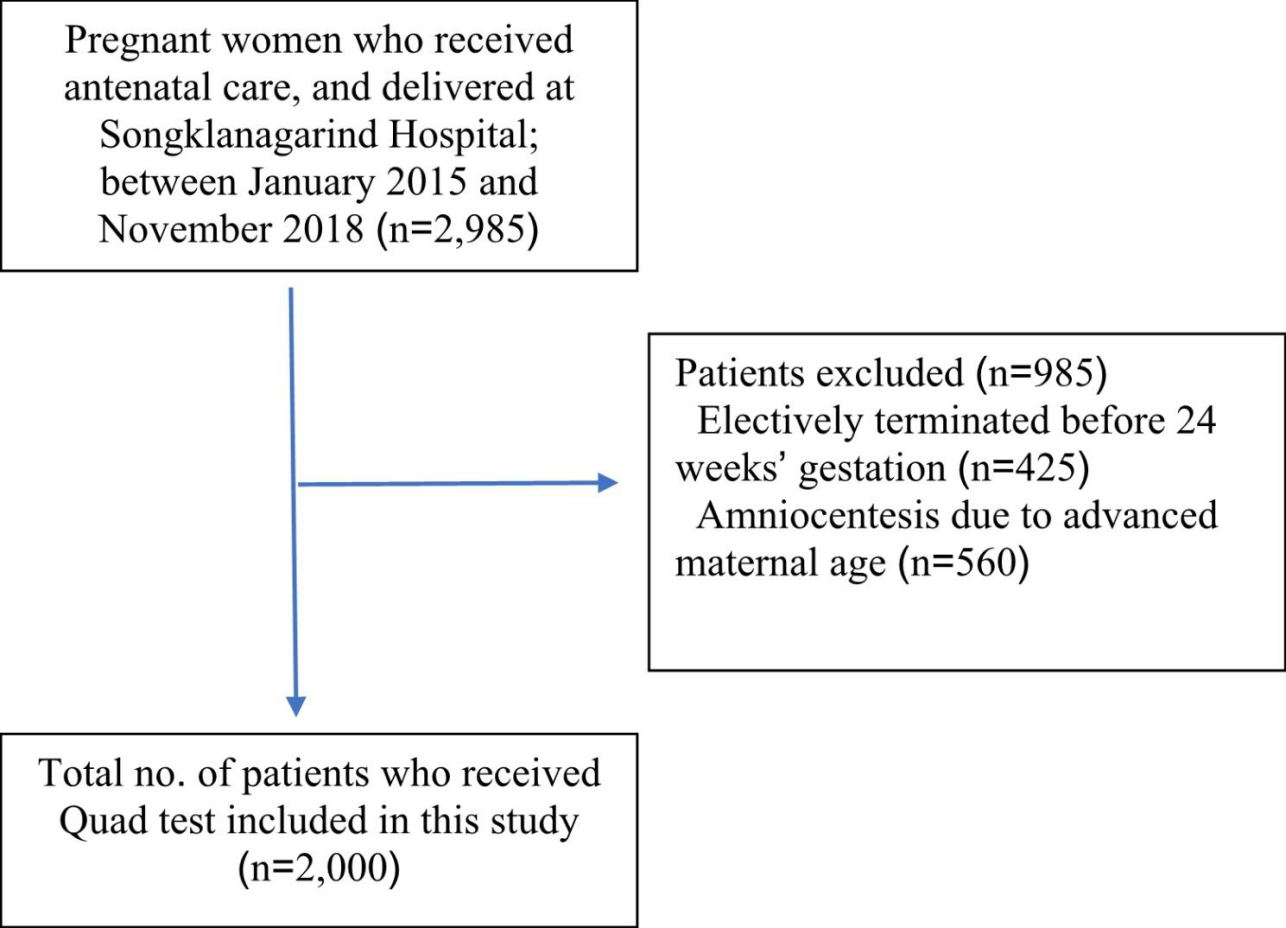
Identification of enrolled patients

This retrospective cohort study was conducted between January 2015 and November 2018 in Songklanagarind Hospital. This study received approval from the Human Research Ethics Committee of the Faculty of Medicine at Prince of Songkla University (REC.63-004-12-4). The ethics committee (Human Research Ethics Committee of the Faculty of Medicine, Prince of Songkla University) waived the requirement for informed consent to participate. We reviewed the medical records of all pregnant women who underwent the Quad test for Down syndrome screening at 15^+ 0^–20^+ 6^ weeks’ gestation by extracting data from the hospital’s database of computerized medical records. Pregnancies that were electively terminated at less than 24 weeks’ gestation, such as those with serious maternal diseases or an abnormal fetus, were excluded (Fig. 1).

Preeclampsia was diagnosed according to the ACOG criteria [40], defined as hypertension and proteinuria diagnosed after 20 weeks of gestation with a systolic blood pressure ≥ 140 mmHg and a diastolic blood pressure ≥ 90 mmHg in two separate measurements at least 4–6 h apart. Preeclampsia with severe features consisted of (1) systolic blood pressure > 160 mmHg or diastolic pressure > 110 mmHg, (2) serum creatinine > 1.1 mg/dl, (3) thrombocytopenia (platelets < 100,000/µL), (4) serum transaminase twice the normal concentration, (5) persistent headache or other cerebral or visual disturbance, (6) persistent epigastric pain, and (7) pulmonary edema. Late-onset PE was defined as onset at more than 34 weeks’ gestation.

Our model to predict late-onset preeclampsia with severe features was developed using maternal baseline characteristics and the values of the four serum markers assessed in the Quad test. All maternal factors thought to be associated with PE were recorded [22–24], including maternal age, gravidity, parity, gestational age at delivery, body mass index (BMI), history of preeclampsia, history of infertility, history of gestational diabetes mellitus (GDM), history of abortion, previous history of preterm birth, previous history of fetal growth restriction, underlying disease (e.g., chronic hypertension, diabetes mellitus, thyroid, and cardiac disease), as well as antepartum complications such as gestational diabetes mellitus, and fetal growth restriction. The values of maternal serum AFP, hCG, uE3, and inhibin A were measured as multiples of the median (MoM).

Statistical analysis was performed using R software v. 4.1.1 and STATA release 14.2 (Prince of Songkla University, Thailand). All demographic data were summarized and compared between PE and non-PE patients. Differences between the groups were evaluated using the chi-square (χ^2^) test or rank sum test. A receiver operating characteristic (ROC) curve was constructed and the area under the curve (AUC) was examined for each variable’s ability to predict PE. To develop a predictive model for late-onset PE with severe features, all maternal characteristics and median MoM values of the serum Quad test were entered into a logistic regression model in R software. The best-fitting model, as determined by the minimum value of the Akaike Information Criterion, was extracted. The model was then further reduced by sequentially removing variables that had only weak evidence (*p* > 0.05) of association with late-onset PE with severe features, as determined by the change of log-likelihood. The scores were integrated by coefficients of this model. Each predictor level was assigned an integer value score so that the ratios of the scores approximated those among of the coefficients. We then constructed an overall score to predict late-onset PE with severe features from the summary of the individual variable level score.

## Results

**Fig. 2:**
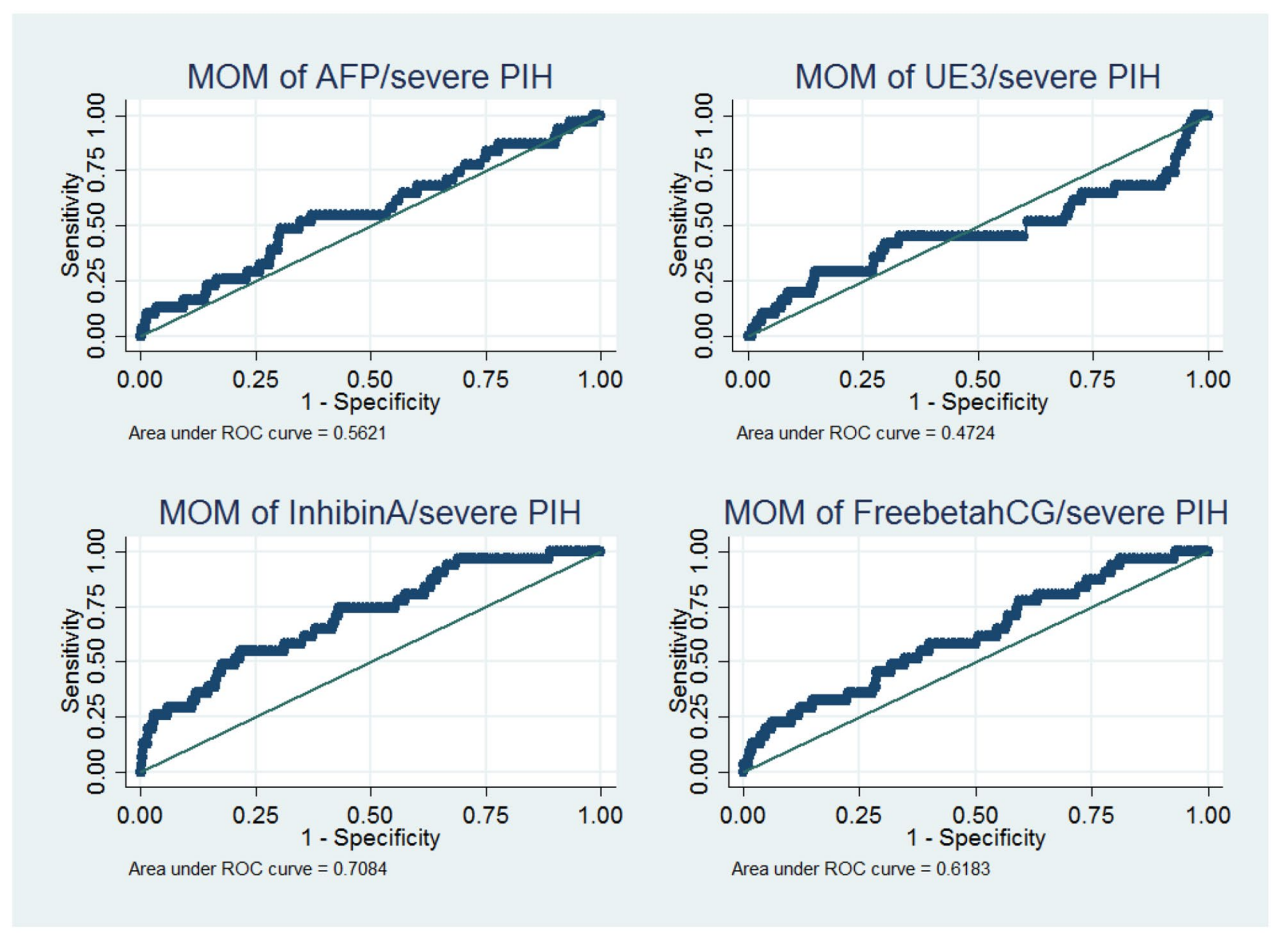
The ROC curve of MoM of four markers for predicting late-onset preeclampsia with severe features (total number of cases = 31) ROC = receiver operating characteristics, MoM = multiple of median

Of the 2000 pregnant women enrolled, 55 (2.75%) developed PE, of whom 31 had late-onset PE with severe features. The median gestational age at delivery was 37.3 weeks (interquartile range: 35.6–38.4). A comparison of baseline clinical characteristics showed that the PE group had significantly higher BMI; earlier gestational age at delivery; longer hospital stay; and a higher rate of history of preeclampsia, history of GDM, previous preterm birth, previous fetal growth restriction, chronic hypertension, diabetes mellitus, renal disease, thyroid disease, GDM, fetal growth restriction, and preterm labor than the non-PE group (Table 1). Furthermore, a higher percentage of perinatal morbidities was observed in the PE group than in the non-PE group (*p* < 0.001). Among the four serum markers of the Quad test, only hCG and inhibin A were significantly associated with PE (Table 1), with inhibin A having the highest AUC in the ROC for predicting late-onset PE with severe features (Fig. 2).

**Table 1:** Characteristics and median MoM values of the Quadruple test

	Preeclampsia n = 55	Non-preeclampsia n = 1945	p-value
**Maternal characteristic** (Median, IQR)			
Maternal age, year	31.5 (28,34)	30 (28,33)	0.061
BMI (Kg/m^2^)	25.1 (22.6,26.8)	22 (19.8,24.7)	< 0.001*
Gravidity	2 (1,2)	2 (1,2)	0.422
Parity	0 (0,1)	0 (0,1)	0.467
Gestational age at delivery, weeks	37.3 (35.6,38.4)	38.7 (38,39.7)	< 0.001*
Date of blood specimen collection	116 (112,120)	117 (111,121)	0.758
Hospital stays	5 (4,6)	4 (3,4)	< 0.001*
**Obstetric characteristic** (Percent)			
Family history of preeclampsia	0 (0.00)	1 (0.05)	1
History of preeclampsia	10 (18.18)	12 (0.61)	< 0.001*
History of infertility	4 (7.27)	12 (0.61)	0.077
History of GDM	6 (10.90)	12 (0.61)	< 0.001*
History of abortion	13(23.63)	368(18.90)	0.481
Previous preterm	4 (7.27)	21 (1.07)	< 0.001*
Previous fetal growth restriction	2 (3.636)	1 (0.05)	< 0.001*
Chronic hypertension	11 (20.00)	26 (1.33)	< 0.001*
Diabetes mellitus	3 (5.45)	3 (0.15)	< 0.001*
Renal disease	2 (3.63)	1 (0.05)	< 0.001*
Thyroid disease	4 (7.27)	27 (1.39)	0.003*
Cardiac disease	2 (3.63)	14 (0.72)	0.105
Autoimmune disease (SLE, APS)	1 (1.88)	7 (0.35)	0.545
GDM	13 (23.63)	145 (7.45)	< 0.001*
Fetal growth restriction	7 (12.72)	11 (0.56)	< 0.001*
Preterm labor	13 (23.64)	126 (6.45)	< 0.001*
**Neonatal outcome** (Percent)			
Neonatal weight (median, IQR (g))	2760 (2272,3389)	3110 (2842,3380)	< 0.001*
1-min Apgar score			< 0.001*
≤ 5	3 (5.45)	40 (2.06)	
> 5	52 (94.55)	1905 (97.94)	
5-min Apgar score			
≤ 5	2 (3.64)	9 (0.46)	
> 5	53 (96.36)	1936 (99.54)	
Admission to the NICU	9 (16.36)	32 (1.65)	< 0.001*
**Median MoM values of the serum markers** (Median, IQR)			
MSAFP	1.1 (0.8,1.2)	1 (0.8,1.2)	0.149
uE3	1 (0.7,1.6)	1.1 (0.8,1.4)	0.849
hCG	1.1 (0.7,1.8)	0.9 (0.6,1.4)	0.046
Inhibin A	1.2 (0.8,1.9)	1 (0.6,1.3)	< 0.001*

*Statistically significant

IQR = interquartile range, MoM = multiple of median, MSAFP = maternal serum alpha-fetoprotein, uE3 = unconjugated estriol, hCG = human chorionic gonadotrophin

**Fig. 3:**
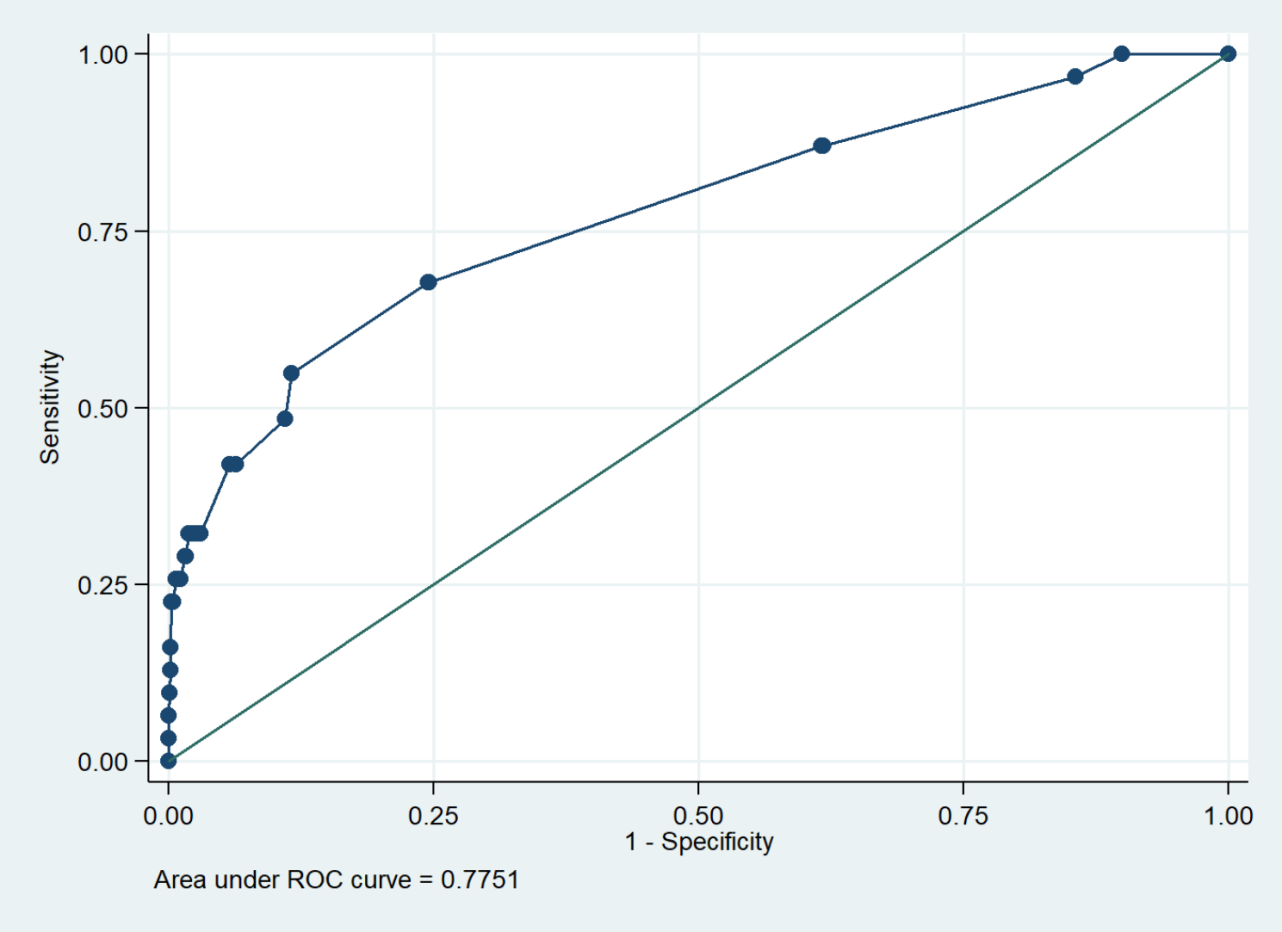
ROC curve of combination between clinical characteristics and MoM of inhibin A for predicting late-onset preeclampsia, with severe features (total number of cases = 31) ROC = receiver operating characteristics, MoM = multiple of median

Table 2 shows the multivariate analysis of the factors associated with late-onset PE with severe features. The significant factors were maternal age ≥ 35 years, history of preeclampsia, history of infertility, cardiac disease, chronic hypertension, thyroid disease, and an inhibin A. Pregnant women with a history of infertility and who received infertility treatment had the highest odds ratio (OR), at 17.50 (95% CI 3.47–88.33), for PE. In particular, pregnant women with thyroid diseases (one patient was hyperthyroid and three were hypothyroid) had increased risk for late-onset PE with severe features (OR 4.89, 95% CI 1.02–23.36). From the logistic regression model, the ROC curve of the combination of maternal factors and MoM of inhibin A was 0.775 for predicting late-onset preeclampsia with severe features (Fig. 3). The score allocation derived from the multivariable logistic regression analysis (Table 3) was used to predict late-onset PE with severe features. The scores were classified into three levels (Table 4). The likelihood ratio to predict late-onset PE with severe features was 49.4 (total score > 60).

**Table 2:** Predictors of late-onset preeclampsia with severe features

	Odds ratio	95% confidence interval	
Inhibin A ≥ 0.5–≤1 MoM	2.48	0.30,	20.66
Inhibin A > 1–≤2 MoM	4.82	0.62,	37.57
Inhibin A > 2 MoM	14.36	1.73,	119.16
Maternal age ≥ 35	2.12	0.76,	5.88
History of preeclampsia	2.43	0.48,	122.34
History of infertile	17.50	3.47,	88.33
Cardiac disease	9.41	1.58,	59.98
Chronic hypertension	13.55	4.38,	41.90
Thyroid disease	4.89	1.02,	23.36

**Table 3:** Summary of the individual variables level scores for predicting late-onset preeclampsia with severe features

	Coefficient	Score^*^
Inhibin A ≥ 0.5–≤1 MoM	0.91	12
Inhibin A > 1–≤2 MoM	1.57	20
Inhibin A > 2 MoM	2.66	35
Maternal age ≥ 35	0.75	10
History of preeclampsia	0.89	12
History of infertile	2.86	37
Cardiac disease	2.24	29
Chronic hypertension	2.61	34
Thyroid disease	1.59	21

*Each score has the value 0 unless specified in the table. Scores derived from the coefficient multiplied by 13 and rounded to the nearest integer

**Table 4:** Performance of the model for predicting late-onset preeclampsia with severe features

Total Score	Percentage of all patients	Percentage developing late onset PE with severe features (95% CI)	Likelihood ratio
>60	0.8	43.8 (19.4, 68.1)	49.4
30–60	15.0	3.7 (1.5, 5.8)	2.4
<30	84.2	0.8 (0.3, 1.2)	0.5

## Discussion

In this study, late-onset PE patients comprised the majority of PE patients at our institute, and almost all patients were at late preterm to term gestation and still had a higher percentage of perinatal morbidities than non-PE patients. The logistic regression model demonstrated that a combination of maternal risk factors (maternal age, history of preeclampsia, history of infertility, cardiac disease, chronic hypertension, and thyroid disease) and inhibin A levels from the Quad test had a good predictive ability for late-onset PE with severe features.

Late-onset PE has a pathogenesis that is different from that of early-onset PE. Late-onset PE patients usually have a normal placental weight, and the fetuses frequently have appropriate birth weight for gestational age and an umbilical artery Doppler velocimetry generally within normal range [5–7, 41]. Late-onset PE is thought to result from an imbalance between the cardiovascular supply of the pregnant woman and the metabolic demands of the fetuses and their placenta in the third trimester period [6, 42]. Patients who develop late-onset PE had a high BMI, increased cardiac output, and relatively unchanged total vascular resistance [6]. Previous reports have focused on predicting late-onset PE (severe and non-severe) and have reported that maternal risk factors alone result in an overall detection rate of 33.5–62.9% [20, 27] using the criteria proposed by the NICE and ACOG. The detection rate was slightly improved when combined with Doppler ultrasound, serum pregnancy-associated plasma protein A, and placental growth factor in the first trimester gestation [20]. Although these combined tests might have a high detection rate for late-onset PE, these are often beyond the capacity of health services in developing countries. Our study was specifically aimed at predicting late-onset PE with severe features, which, to our knowledge, has not been reported in any previous work, and showed that not all maternal risk factors of PE increased the risk of late-onset PE with severe features.

This study found that maternal age, history of PE, history of infertility, cardiac disease, chronic hypertension, and thyroid disease were all significant risk factors for late-onset PE with severe features. Pregnant women with a history of infertility and who underwent infertility treatment were found to have the highest risk for PE. Previous studies [43–46] have also reported that infertility status and infertility treatment confer an increased risk of hypertensive disorders during pregnancy. In addition to advanced age, high estradiol levels in the IVF cycles potentially increase the risk for PE due to abnormal placentation and ultimately uteroplacental vascular insufficiency in infertile pregnant women [44, 45]. Furthermore, a recent report showed a comorbid relationship between infertility and other pathologies and highlighted shared genes and molecular pathways related to hypertensive disorders in pregnant women [43]. We found that pregnant women with thyroid diseases had increased risk for late-onset PE with severe features, despite receiving treatment and having no symptoms of thyroid dysfunction. Previous reports [47, 48] have also found that thyroid dysfunction, especially hypothyroidism, is associated with risk of PE, which had increase in endothelial cell dysfunction. In addition to some specific maternal risk factors related to late-onset PE with severe features, we found that serum inhibin A during the second trimester had an additive value for improving the prediction of PE.

Inhibin A is a glycoprotein hormone of the transforming growth factor-β superfamily produced by the placenta during pregnancy [48]. Inhibin A can regulate embryo implantation and differentiation; affect the normal permeability, integrity of maternal blood vessels, and adaptability of the maternal cardiovascular system to pregnancy; reduce placental blood flow; and aggravate placental ischemia and metabolic disorders [49]. Previous reports [33–39] have reported an association between developing preeclampsia, especially in early-onset type [35, 37, 50], and a high inhibin A level. A systematic review demonstrated that the best predictor for PE among pregnant women who underwent the Quad test for Down syndrome screening was inhibin A, with a positive likelihood ratio of 19.52 (95%CI 8.33–45.79) [34]. Our study revealed that inhibin A had the highest AUC (0.708) for predicting late-onset PE with severe features, and when we combined inhibin A with maternal risk factors, the AUC improved to 0.775, with a high predictive value among patients with a score > 60. This predictive model with both maternal risk factors and inhibin A also had a higher predictive ability than the model using maternal risk factors alone, proposed by the NICE and ACOG guidelines (AUC 0.591 [0.538–0.644] and 0.695 [0.604–0.714], respectively), in predicting any PE at < 37 weeks’ gestation in the Asian population [27].

According to the pathogenesis, late-onset PE is different from early-onset PE, which is a defective placentation in origin, so as the use of low-dose aspirin might have a limited value for prevention of this condition [23, 24]. However, patients that develop late-onset PE have a high BMI, increased cardiac output, and relatively unchanged total vascular resistance [6]; therefore, some researchers are also interested in behavioral modifications to prevent PE. There is evidence that a healthy diet, appropriate weight gain, exercise, and stress reduction can reduce the risk of PE, without significant differences in birth weight or small for gestational age [51, 52]. Therefore, lifestyle modifications should be recommended for patients who have a risk of late-onset PE (for primary prevention purposes). Furthermore, the monitored blood pressure and abnormal symptoms by themself in the third trimester period are also suggested for early detection and diagnosis (especially severe features) in order to reduce maternal morbidities and mortality (for secondary prevention purposes). Currently, there is an increase in ongoing research that focus on new drugs for pregnant woman during late second to early thrid trimester periods, such as pravastatin, proton-pump inhibitors, and metformin, that act against both placental and maternal vascular diseases; which can reduce circulating soluble fms-like tyrosine kinase-1 (sFlt-1) and up-regulate nitric oxide synthase. These reduce antiangiogenic factors, and the proinflammatory state might have an effect on prevention and treatment of late-onset PE patients [53].

A strength of our study is that it is the first study to develop a model of predictive risk factors for late-onset PE with severe features using both maternal risk factors and the Quad test in pregnant women in the second trimester with a good predictive ability. This predictive model would benefit patients by resulting in close monitoring of those at a high risk of developing late-onset PE with severe features and assist in early diagnosis in order to reduce maternal morbidities and mortality from this condition, particularly in developing countries where the majority of pregnant women usually only receive antenatal care in the second trimester. However, an important limitation of our study was its retrospective design and limited number of patients who may have had other maternal risk factors that might be associated with this condition.

## Conclusions

In conclusion, a model combining serum inhibin A with maternal risk factors was useful in predicting late-onset PE with severe features. Therefore, close monitoring of these high-risk patients for PE is recommended for the early diagnosis and secondary prevention of maternal morbidity and mortality related to PE.

(total number of cases = 31)

## Data Availability

The datasets used and/or analysed during the current study available from the corresponding author on reasonable request.

## Abbreviations

ACOG: The American College of Obstetricians and Gynecologists
AFP: Alpha Fetoprotein
AUC: Area under the curve
BMI: Body mass index
FIGO: Federation of Gynecology and Obstetrics
GDM: Gestational diabetes mellitus
hCG: Human chorionic gonadotropin
MoM: Multiples of the median
NICE: National Institute for Health and Care Excellence
PE: Preeclampsia
ROC: Receiver operating characteristic
uE3: Unconjugated estriol
